# Enhancement stimulants: perceived motivational and cognitive advantages

**DOI:** 10.3389/fnins.2013.00198

**Published:** 2013-10-31

**Authors:** Irena P. Ilieva, Martha J. Farah

**Affiliations:** Department of Psychology, Center for Neuroscience and Society, University of PennsylvaniaPhiladelphia, PA, USA

**Keywords:** cognitive enhancement, prescription stimulants, Adderall, Ritalin, motivation

## Abstract

Psychostimulants like Adderall and Ritalin are widely used for cognitive enhancement by people without ADHD, although the empirical literature has shown little conclusive evidence for effectiveness in this population. This paper explores one potential explanation of this discrepancy: the possibility that the benefit from enhancement stimulants is at least in part motivational, rather than purely cognitive. We review relevant laboratory, survey, and interview research and present the results of a new survey of enhancement users with the goal of comparing perceived cognitive and motivational effects. These users perceived stimulant effects on motivationally-related factors, especially “*energy*” and “*motivation*,” and reported motivational effects to be at least as pronounced as cognitive effects, including the effects on “*attention*.”

## Introduction

Stimulant medications such as amphetamine and methyphenidate have long been used by healthy individuals to enhance work performance (see Rasmussen, 2008, for a history). These medications are currently widely used as study aids by college students in the US and Canada (Poulin, 2007; Smith and Farah, 2011) and, to a lesser extent, in many other countries (Sahakian and Morein-Zamir, 2007; Franke, 2011; Castaldi et al., 2012; Partridge et al., 2013), providing a non-hypothetical case in point for neuroethical analyses of cognitive enhancement. It is therefore surprising that a growing number of researchers now question whether these medications do, in fact, enhance cognition (for reviews, see Advokat, 2010; Chamberlain et al., 2010; Hall and Lucke, 2010; Repantis et al., 2010; Smith and Farah, 2011). In particular, comprehensive reviews of the literature on stimulants' effects on healthy cognition have noted that there is “very weak evidence that putatively neuroenhancing pharmaceuticals in fact enhance cognitive function.” (Hall and Lucke, 2010), even proposing “that stimulants may actually impair performance on tasks that require adaptation, flexibility and planning” (Advokat, 2010). We carried out a double-blind, placebo-controlled study on the effects of mixed amphetamine salts (Adderall), which was adequately powered to find medium effects. Based on our own failure to find a single drug effect across numerous measures of executive functions, memory, creativity, intelligence, and standardized test performance, we concluded that Adderall “has no more than small effects on cognition in healthy young adults” (Ilieva et al., 2013).

This raises the question of why people persist in using stimulants to enhance schoolwork and, according to anecdotal evidence, other cognitively demanding duties such as stock trading, entrepreneurship, surgery, and professional academic work (Sahakian and Morein-Zamir, 2007; Franke et al., 2013; Kolker, 2013). It may be that a small cognitive advantage is useful in these situations and that is all users seek with continued use. Alternatively, it may be that individual differences result in sizeable cognitive advantages for some users, and they are the ones who use regularly. A third possibility is that users gain a *non-cognitive* advantage that helps them perform better in school and on the job. This is the alternative to be explored in this review.

### Stimulants, cognition, and motivation

The question of whether stimulant-related positive affective states would impact the performance of cognitive tasks implies a distinction between motivation and cognition and the role for each in work performance. Because these terms have received various definitions in the past, we begin by specifying the sense in which we use them in the present paper.

By *cognition* we mean the processes of encoding, storing, and manipulating information. Attention, memory, and executive function are examples of cognitive processes by this general definition. By cognitive ability we therefore mean the ability to carry out these processes, which varies among normal healthy people.

We will use the term *motivation* to refer to a similarly broad set of affective states that influence whether a person will voluntarily use their cognitive ability in the performance of a task. By this general definition, a number of factors (which often, but not always, co-occur) reflect or contribute to task motivation: namely, wanting to complete a task, enjoying it or being interested in it. Motivation may also be supported by closely related factors, such as positive mood, alertness, energy, and the absence of anxiety. Although motivation is a state, there are trait-like differences in the motivational states that people typically bring to tasks, just as there are differences in cognitive ability.

These definitions of cognition and motivation correspond to the “can't/won't” distinction. The performance of cognitively demanding tasks are subject to limitations of cognitive ability, as when a subject can't perform beyond a certain level, and limitations of motivation, as when a subject won't make the effort to perform beyond a certain level. This distinction is admittedly somewhat vague and intuitive, rather than precise and analytic, but we know of no better way of distinguishing between cognition and motivation in this context.

Objective evidence that cognitive ability is only minimally enhanced by stimulants comes from tasks in which motivation is minimally taxed, as these tasks are typically tedious and rewarded for completion (e.g., in a representative study from our lab, participants completed about 2 h of neuropsychological tests, such as Flanker, Go/No-Go, and NBack, while being compensated uniformly regardless of performance, Ilieva et al., 2013). It is possible that stimulants enhance schoolwork and other cognitively demanding work in everyday contexts where there is larger room for motivated work: where tasks are more intellectually engaging, where reward depends on performance, and where outcomes determine users' future employability and reputation. To explore here the prediction that users view stimulants' benefits on motivation as equal or greater than those on cognition, we begin by reviewing the laboratory literature on stimulants' effects, as well as enhancement users' self-reported reasons for using stimulants in real-world settings.

### Stimulant effects on motivation: laboratory and clinical considerations

Animal research provides ample evidence that the mesolimbic dopamine system is central to motivation (Koob, 1996; Berridge, 2007; Faure et al., 2008) and stimulant medications are known to increase activity in this system (Butcher et al., 1988; Drevets et al., 2001; Volkow et al., 2004). The first psychological effects of these drugs noted by researchers were increased energy, drive, enjoyment, and motivation (Rasmussen, 2008). Stimulants are currently used to treat apathy in neurological and psychiatric patients (Roth et al., 2007). On the basis of these facts one would expect an effect of stimulants on motivation in normal, healthy humans performing cognitive tasks. Although relatively little research has investigated this directly, the existing evidence is consistent with this expectation.

Wardle and de Wit (2012) studied the effect of amphetamine on task enjoyment. Healthy normal subjects were shown photographs from the International Affective Pictures set and asked to rate their reactions to the stimuli in terms of emotional valence and degree of arousal. The drug increased subjects' enjoyment of all pictures, as assessed by self-report. For positive pictures, subjects also showed enhanced enjoyment by a decreased EMG-measured reaction of the corrugator (“frown”) muscle and elevated reaction of the zygomatic (“smile”) muscle. Thus, these findings corroborated stimulants' effect on increased liking for task-related material, a factor contributing to motivation.

Another way to operationalize motivation is through the amount of work invested to obtain a reward. A recent double-blind placebo-controlled study asked whether amphetamine increased the expenditure of effort for reward. Participants were given a sequence of choices between a high-effort task promising large monetary compensation and a less effortful, less profitable task. The low-effort task required participants to push a button repeatedly with their index finger, while the high-effort task entailed a longer duration of button-pressing, using one's pinky finger. The probability of reward also varied across trials, with the result that some trials offered an uncertain chance of high reward for high effort, thus demanding high motivation. Amphetamine increased the proportion of high reward/high effort choices. These results imply that amphetamine may enhance the motivation to work for uncertain rewards where the lack of guarantee of reward would be expected to tax motivation maximally (Wardle et al., 2011).

Finally, several research groups have noted that subjects find stimulants particularly rewarding when combined with performance of a cognitive task compared to the stimulant with no cognitive task or the cognitive task without a stimulant. For example, after trying amphetamine and a benzodiazepine, subjects chose to combine amphetamine with an attention task but not with a relaxation task (Silverman et al., 1994a). Subjects rated a mathematical task as more “interesting” when performed with methylphenidate, and showed disproportionate striatal dopamine release when math was performed with the drug, compared to no math or no drug (Volkow et al., 2004). Finally, subjects were more willing to work to earn methylphenidate for use while performing a math task, compared to for use during a relaxation session (Stoops et al., 2005). Similar findings have been obtained with caffeine (Silverman et al., 1994b) and the novel stimulant modafinil (Stoops et al., 2005).

### Motivational effects in enhancement uses of stimulants

The question arises whether stimulants' motivation-elevating influence remains robust in real-world contexts, where the setting and nature of work may be very different from laboratory tasks of motivation.

Most evidence on this issue is indirect. The role of non-cognitive factors in the use of stimulants for enhancement is hinted at by the elevated rates of depression and anxiety among students who use them (e.g., Weyandt et al., 2009; Rabiner et al., 2010; Teter et al., 2010; Dussault and Weyandt, 2013). This is consistent with students self-medicating for psychological distress or apathy in order to overcome emotional barriers to work performance. Of course, the causal relations among depression, academic performance, and stimulant use are likely to be complex (Ford and Schroeder, 2008) rendering these findings only suggestive at best.

Surveys of students' reasons for using stimulants also indicate a role for non-cognitive factors, but the multiple-choice alternatives offered have not been designed to separate the roles of cognitive and motivational effects in academic performance enhancement. Rather, they probe for reasons related to socializing, recreational drug usage and academic concerns, without distinguishing the roles of cognitive and motivational effects on this last category of reasons. Results indicate that students are sensitive to the non-cognitive effects and exploit them outside of schoolwork. For example, students report such reasons as getting “high” and having “fun” (Teter et al., 2005, 2006; Boyd et al., 2006; DeSantis et al., 2008, 2009; Judson and Langdon, 2009; Rabiner et al., 2009; Clegg-kraynok et al., 2011; Dussault and Weyandt, 2013). Students note mood-elevating potential of stimulants (Carroll et al., 2006; Rabiner et al., 2009). When the surveys include response choices such as “energy” or “alertness” in their list of reasons for using stimulants, high response rates for these items are found (e.g., Hall et al., 2005; Teter et al., 2005, 2006; Boyd et al., 2006; Carroll et al., 2006; Judson and Langdon, 2009; Clegg-kraynok et al., 2011; Peterkin et al., 2011; Dussault and Weyandt, 2013). Two surveys included one item each that directly probed motivational effects in relation to schoolwork: DeSantis et al. (2008) included “to make work more interesting” and Bavarian et al. (2013) included “to make studying more enjoyable” in their list of reasons and found 12 and 58% of their respondents, respectively, endorsed these items.

Vrecko (2013) took a different approach to understanding students' reasons for using stimulants as study aids, conducting open-ended interviews with 24 university students and former students who identified themselves as users of Adderall for academic enhancement. Qualitative data analyses showed that users highlighted stimulants' positive influence on mood, energy, and other motivation-related states. Typical statements were “[on Adderall] I didn't want to stop what I was doing until it was completed up to a certain level of my satisfaction,” and “You're interested in what you're doing even if it's boring.” One respondent summed up the cognitive vs. motivational effect this way: “Adderall doesn't necessarily make you smarter […], the main benefit, really, is that on it, I don't mind doing work.” Vrecko concluded that “alteration of emotions appears to be an important dimension of the drug effects that users perceive to enable improved academic performance.”

In sum, while stimulants' objectively measured effects on cognition are small, users tend to report substantial perceived enhancement effects. In this paper we call into question the assumption that these sizeable perceived effects are purely cognitive. Evidence from many sources is consistent with the hypothesis that the motivational effects of stimulants are at least as important as the cognitive effects in enhancing students' academic performance. However, with the exception of Vrecko's qualitative research, this evidence comes from research with animals and clinical populations and simple laboratory tasks, or from surveys containing just one relevant response option. No study has directly compared the perceived motivational advantage of these medications to their perceived cognitive benefit. To do so, we probe a number of cognitive and motivational constructs and quantify users' ratings of stimulants' ability to enhance them.

Hence, the purpose of the present study was to test, quantitatively, the prediction that enhancement users experience substantial motivational advantages from stimulants and that these effects are as pronounced as or more pronounced than the cognitive advantages. College student stimulant users without ADHD were asked to rate the magnitude of enhancement drugs' perceived effects on various aspects of motivation and cognition. Our focus was on the magnitude of the reported motivational advantages, especially in comparison to the perceived cognitive effects, previously assumed to be the major driver of use.

## Methods

### Participants

Participants were 40 University of Pennsylvania undergraduates with no self-reported history of ADHD who had used enhancement stimulants at least once in their lives^1^. Specifically, 10 participants had used a stimulant for enhancement just once, 5 on 2 occasions, 7 on 3–5 occasions, 7 on 6–9 occasions, 4 on 10–19 occasions, 2 on 20–39 occasions, and 5 on 40 or more occasions. Participants were recruited from psychology classes in exchange for course credit and agreed to participate in a larger survey ostensibly exploring students' academic behaviors and beliefs. One hundred and seventy-one potential participants were screened out of the survey as non-users of enhancement stimulants.

### Procedure and materials

Participants completed an online survey that assessed past enhancement use and stimulants' perceived effects^2^. Enhancement use was measured as the number of occasions of use in the past month, past academic year, and in one's lifetime. The specific prompt, adapted from Teter et al. (2010), read as follows: “On how many occasions have you used ADHD medication (e.g., Adderall, Ritalin, or other), *without* a prescription, to help you do well at school and/or work?”

Students were then asked to rate how helpful they found ADHD medications for 14 different psychological functions chosen from popular and scholarly descriptions of cognitive enhancement with stimulants. They indicated their answers on a 7-pont scale from *Very Impairing* to *Very Helpful* (where the midpoint referred to *No Effect*). Half of the functions presented were purely cognitive, that is functions that concerned cognitive abilities and did not carry any implication about an individual's affective state. These were: *focused attention, memory, creativity, intelligence, ability to multitask, speed of thinking*, and *abstract thinking*. The other half of the functions were non-cognitive functions related to motivation in that they support task performance by increasing subjective energy and enjoyment and decreasing subjective effort and avoidance: *motivation, task enjoyment, mood, self-confidence, energy, alertness*, and *anxiety relief*. Each participant saw these items in a different randomized sequence.

## Results

The main goal of data analysis was to assess enhancement users' perceptions of the motivational effects of stimulant medications. Analyses therefore focused on ratings of the medications' effects on non-cognitive, motivation-related functions. In particular, we assessed: (1) whether users reported positive effects on motivation-related functions; (2) whether this overall perception of motivational enhancement was greater than, less than, or equivalent to the overall perception of cognitive enhancement in the narrow sense, assessed by the cognition-related ratings; and (3) which particular functions within each category were believed to be significantly enhanced by these users.

Participants found that stimulants were, overall, enhancing of both motivation-related and cognition-related functions. Linear composite ratings in each category, with all individual constituent functions equally weighed, indicated helpfulness (i.e., ratings exceeded “no effect” by one sample *t*-tests): *t*_(39)_ = 11.17, *p* < 0.01 and *t*_(39)_ = 8.56, *p* < 0.01, respectively, two-tailed^3^.

In a comparison between the enhancing effects of stimulants on linear composites of motivation and cognition, participants perceived greater enhancement of functions in the motivational category, paired-samples *t*_(39)_ = 2.19, *p* = 0.04, two-tailed. In sum, for the sample of functions used in this project, enhancement users of stimulants found that the pills enhanced motivation, and indeed reported that the pills enhanced motivation significantly more than cognitive ability.

Turning to the individual functions rated, Table 1 shows the means of the perceived stimulant effects on each function within the two categories. A cognitive function (*attention*) was rated numerically most strongly enhanced by stimulants, but it was not rated significantly higher than the next three in descending order: energy, alertness, and motivation, by paired-samples *t*-tests: [*t*_(39)_ = 0.82, *p* = 0.42; *t*_(39)_ = 0.77, *p* = 0.45; and *t*_(39)_ = 0.85, *p* = 0.40, respectively]. Equivalence testing, with differences <0.5 points on the 7-point scale stipulated to be equivalent, suggested that the standard 90% confidence interval (Walker and Nowacki, 2011) of the difference between the variables ([−0.13, 0.38], for the *attention-energy* contrast; [−0.18; 0.48], for the *attention-alertness* contrast; [−0.15, 0.45], for the *attention-motivation* contrast) fell within the equivalence interval of [−0.50; 0.50]. Taken together, null-hypothesis and equivalence tests converged to suggest that attention ratings were comparable to the ratings on the three highest rated motivation-related functions.

**Table 1:** **Perceived stimulant enhancement effects on motivation and cognition**.

	**Descriptives**			**One-sample *t*-tests**			
	***N***	Mean perceived effect^*^	**Std. Dev.**	***t***	***df***	**Sig. (2-tailed)**	**Mean difference from “no effect”**
**COGNITION-RELATED FUNCTIONS**							
Ability to multitask	40	4.47	1.826	1.646	39	0.108	0.475
Abstract thinking	40	4.75	1.276	3.717	39	0.001	0.750
Attention	40	6.30	1.067	13.633	39	0.000	2.300
Creativity	40	4.45	1.154	2.467	39	0.018	0.450
Intelligence	40	4.98	1.271	4.853	39	0.000	0.975
Memory	40	5.15	1.099	6.618	39	0.000	1.150
Speed of thinking	40	5.78	1.310	8.567	39	0.000	1.775
**MOTIVATION-RELATED FUNCTIONS**							
Alertness	40	6.15	0.864	15.742	39	0.000	2.150
Anxiety relief	40	4.13	1.522	0.519	39	0.606	0.125
Energy	40	6.18	0.874	15.743	39	0.000	2.175
Mood	40	4.78	1.476	3.321	39	0.002	0.775
Motivation	40	6.15	1.075	12.645	39	0.000	2.150
Self-confidence	40	5.13	1.539	4.623	39	0.000	1.125
Task enjoyment	40	5.40	1.482	5.977	39	0.000	1.400

* 7, Extremely helpful; 6, Very helpful; 5, Somewhat helpful; 4, No effect; 3, Somewhat impairing; 2, Very impairing; 1, Extremely impairing.

Considering all of the functions (motivational and cognitive) examined, most but not all were rated as significantly enhanced, 12 of the 14 ratings of enhancement significantly differed from “no effect” using a series of one-sample *t*-tests (Table 1). Correcting for multiple comparisons by dividing the critical *p*-value of 0.05 by 14, 11 functions remain rated as significantly enhanced by this more appropriately conservative criterion. These included 6 motivational functions and 5 cognitive functions.

These findings remained generally unchanged after (a) exclusion of 2 participants with univariate outlier ratings falling more than 3 SD away from the mean of the other 40 participants of the sample and (b) after exclusion of the subsample of 10 participants who used stimulants for enhancement only once in their lifetime^4^.

## Discussion

There is an emerging consensus in the literature on cognitive enhancement that the cognitive benefits of prescription stimulants are modest. The most commonly used medications for cognitive enhancement, amphetamine and methylphenidate, seem to have limited effects on laboratory measures of executive function and learning in normal, healthy young adults. Nevertheless, these drugs are widely used to enhance work performance by college students and others engaged in cognitively demanding work. In the present article we explored the possibility that these drugs are used to enhance motivation rather than, or in addition to, cognition.

Recent laboratory evidence suggests that amphetamine and methylphenidate enhance motivation-related processes in healthy participants, and the in-depth interviewing conducted by Vrecko (2013) indicates that enhancement users find the motivational effects of these drugs helpful for enhancing schoolwork. Here we add to the laboratory, survey, and interview evidence by performing the first survey directly comparing users' ratings of stimulants' motivation- and cognition-enhancing effects.

The present study found that student users perceive stimulants as beneficial for cognition, despite the weak evidence for objective cognitive enhancing effects (see Introduction). However, student users also perceive stimulants as advantageous for motivation. *Motivation*, *energy*, and *attention*, the functions viewed as most strongly enhanced, did not differ in their susceptibility to stimulants' subjective effects. Not only were motivational functions found by users to be significantly enhanced, they were found to be somewhat more enhanced as a group than a category of cognitive functions, and this difference was statistically significant. This finding extends previous knowledge by demonstrating that stimulants' motivational effects are viewed by healthy users as prominent despite the common assumption that they work chiefly on cognition. Although our study is limited to assessing *perceived* enhancement and does not speak to actual behavioral effects on motivation, cognitive processing, or task performance, the results document experiential effects relevant to users' decisions to practice enhancement with prescription stimulants. According to students who use stimulants for cognitive enhancement, these drugs may enable better performance of cognitively demanding work at least in part through their effects on motivation.

These patterns of data emerged despite measurement limitations, including the assessment of enhancement effects over a lengthy period of time, potentially conducive to recall errors in participants' estimates. Future research should address questions unexamined in the present study, including the possible moderation of perceived motivational effects by specific medication (e.g., amphetamine vs. methylphenidate) or dose (high vs. low). Additionally, further research is needed to show whether the motivational effects of these drugs are particularly valuable under conditions of fatigue or sleep deprivation.

The present data support the hypothesis that enhancement users rely on ADHD medication for boosting drive, energy, and mood, rather than cognitive capacity alone. Thus, our study opens up novel avenues for future research. How do individual differences and different types of academic work moderate the effects discussed here? How does stimulant-enhanced motivation affect actual performance? Do the present conclusions, regarding enhancement uses of stimulants, also apply to therapeutic uses in neuropsychiatric disorders? What alternative interventions for improving achievement motivation might be possible, with fewer risks than prescription stimulants?

## Author contributions

Both authors designed and planned the study, analyzed, and interpreted the data and wrote the manuscript. Irena P. Ilieva collected the data.

### Conflict of interest statement

The authors declare that the research was conducted in the absence of any commercial or financial relationships that could be construed as a potential conflict of interest.

## Footnotes

^1^We chose to focus on enhancement use over the lifetime (as opposed to the past month or the past year) because this approach rendered a maximum number of participants eligible, and hence allowed greatest statistical power. Although participants' frequency estimates over this long period may be prone to imprecision, we had no theoretical reason to restrict our sample to past-month or past-year users, potentially omitting useful data from use outside of these periods.

^2^In addition to stimulants' perceived effects, these 40 subjects and the other 171 also answered four other sets of questions. They were intended for both enhancement users and non-users and concerned differences between the groups in achievement motivation, lay theories of intelligence, and media multitasking frequency. Because outside the scope of the present paper, these data are not reported here.

^3^We used two-tailed tests throughout our analyses to avoid overlooking unexpected scenarios, including the possibility that users may view stimulants as impairing to a given ability, or the possibility that, as the neuroethics literature has traditionally suggested, the perceived effects on cognition might outweigh those on motivation.

^4^With the exception that, in (b), no evidence for equivalence emerged for the comparison between ratings of *alertness* and *attention*, and only five functions in the motivation category showed significant perceived enhancement.
